# Hydroquinidine displays a significant anticarcinogenic activity in breast and ovarian cancer cells via inhibiting cell-cycle and stimulating apoptosis

**DOI:** 10.55730/1300-0152.2640

**Published:** 2023-01-11

**Authors:** Mervenur YAVUZ, Betül ŞAHİN, Ahmet Tarık BAYKAL, Turan DEMİRCAN

**Affiliations:** 1 Institute of Health Sciences, Muğla Sıtkı Koçman University, Muğla, Turkey; 2 Acıbadem Labmed Clinical Laboratories, İstanbul, Turkey; 3 Department of Medical Biochemistry, School of Medicine, Acıbadem Mehmet Ali Aydınlar University, İstanbul, Turkey; 4 Department of Medical Biology, School of Medicine, Muğla Sıtkı Koçman University, Muğla, Turkey

**Keywords:** Cancer, hydroquinidine, proteomics, MCF-7, SKOV-3, anticancer agent

## Abstract

Breast and ovarian cancers are women’s most commonly diagnosed cancers. Seeking an efficient anticarcinogenic compound is still a top priority regarding the aggressiveness of these cancers and the limited benefit of current therapies. Hydroquinidine (HQ) is a natural alkaloid used in arrhythmia and Brugada syndrome. As an ion channel blocker, HQ exhibits its activity by altering ion gradient and membrane potential. Considering the growing evidence of ion channel blockers’ antineoplastic potential, we were prompted to test HQ’s effect on breast and ovarian cancers. MCF-7 and SKOV-3 cell lines were used to inspect how HQ acts on survival, clonogenicity, migration, tumorigenicity, proliferation, and apoptosis. The molecular basis for the remarkable antiproliferative and proapoptotic effect of HQ in these cells was dissected by proteomics. CDK1, PSMB5, PSMC2, MCM2, MCM7, YWHAH, YWHAQ, and YWHAB proteins in HQ-treated MCF-7 cells, and RRM2, PSMD2, PSME2, COX2, COX4l1, and CDK6 proteins in HQ-treated SKOV-3 cells were found as low-abundant, which was noteworthy. Based on the in-depth analysis, upon HQ treatment, several cell cycle-related processes were found as suppressed, whereas apoptosis and ferroptosis pathways were found to be activated. The observed proteome alteration in cancer cells may provide mechanistic explanations for the growth-limiting effects of HQ at the cellular level.

## 1. Introduction

Breast cancer (BC) is one of the most common types of cancer in women (Łukasiewicz et al., 2021), and ovarian cancer (OC) is the third most mortal gynecological cancer worldwide according to 2020 Global Cancer Statistics (Huang et al., 2022). Due to the increasing rate of diagnosis and death among women, BC and OC have become severe health problems to solve. The therapy methods implemented in BC treatment include surgery (lumpectomy or mastectomy), chemotherapy, radiation therapy, and systemic therapy (Moo et al., 2018). The current standard therapy methods for OC are surgery and platinum-based chemotherapy (Cortez et al., 2018). Treatment methods can be curative in the early stages; however, as the disease advances, recurrence occurs within the following couple of years (Kurnit et al., 2021) with progressively shorter disease-free survival. Thereby, searching for a drug with robust antineoplastic activity to use in these cancers is highly required.

Ion channels are ubiquitously expressed on the surface of the cell membrane and organelles. In addition to the well-characterized roles in the control of transport, ion concentration gradient, and membrane potential, they are central elements of proliferation, migration, differentiation, and apoptosis by regulating signaling cascades (Chen et al., 1994; Shen et al., 2020). Recent studies unveiled the causative link between alterations in ion channel expression or activity and cancer initiation or progression (Litan and Langhans, 2015; Altamura et al., 2022). Since ion channels act as signal transducers and regulate cellular volume and ion flow, in numerous cancer types they play key roles in cell motility, migration, metastasis, proliferation, angiogenesis, resistance to chemotherapy, and cellular death pathways such as apoptosis and autophagy (Litan and Langhans, 2015; Altamura et al., 2022). Their critical contribution to the manifestation of cancer hallmarks positions them as excellent targets in cancer treatment. Therefore, blocking ion channels to suppress tumor growth is a promising strategy recently gaining momentum.

Hydroquinidine (HQ, a.k.a. Dihydroquinidine), is a cinchona alkaloid that prolongs the duration of QT intervals and prevents ventricular arrhythmias in rare hereditary short QT syndrome (Giustetto et al., 2011). It has also been demonstrated that HQ can be used as an antiarrhythmic agent in sudden cardiac death-related arrhythmogenic Brugada syndrome (Hermida et al., 2004). Studies on HQ indicated that it blocks multiple ion channels, particularly the potassium ones (Mercer et al., 2016; Perrin et al., 2018; El-Battrawy et al., 2019). Although its mode of action as ion channels blocker is well characterized, the complete list of blocked ion channels is yet to be described.

Since misregulation of the level and activity of ion channels has been linked to cancer pathology, and ion channels play a significant role in tumor progression (Xia et al., 2017), evasion of apoptosis (Hoffmann and Lambert, 2014), invasion (Crottès and Jan, 2019), cross-talk with immune cells (Arcangeli, 2011), we speculated that targeting ion channels with HQ in breast and ovarian cancer cells may have an anticancer effect. The putative anticarcinogenic effect of HQ on MCF-7 and SKOV-3 cells was investigated by combining cellular methods and comprehensive proteomics analysis. Our data underlined HQ’s anticancer activity on MCF-7 and SKOV-3 cells, evident in cell-cycle inhibition, apoptosis induction, and a significant decrease in migration capacity plausibly due to the upregulated tumor-suppressor genes and downregulated proto-oncogenes. This pioneering study may expand the toolbox of anticarcinogenic molecules for breast and ovarian cancers.

## 2. Materials and methods

### 2.1. Cell culture maintenance

Ovarian adenocarcinoma (SKOV-3, ATCC Number: HTB-77) and breast adenocarcinoma (MCF-7, ATCC Number: HTB-22) cells were provided from our own stocks. SKOV-3 cells were maintained in Dulbecco’s Modified Eagle Medium (DMEM, Cat no: D6429, Sigma) supplemented with 10% heat-inactivated fetal bovine serum (FBS, Cat no: A4766801, Gibco) and 1% penicillin streptomycin (Pen/Strep, Cat no: 15140-122, Gibco). MCF-7 cells cultured in Eagle’s Minimum Essential Medium (EMEM, Cat no: M4655, Sigma) containing 10% FBS, 1% Pen/Strep, and 0.01 mg/mL human recombinant insulin (Cat no: P07-04300, Pan Biotechnology). All cell lines were maintained in a humidified chamber with 5% CO_2_ at 37 °C. Culture mediums were changed every other day, and cells were passaged when a minimum of 80% confluency was observed. In each experimental design, cells were deattached using 0.25% Trypsin-EDTA (Cat no: 25200056, Gibco) counted via thoma cell counting chamber (Cat no: 075.03.001; Isolab) and 0.4% Trypan Blue Solution (Cat no: 15250061, Thermofisher Scientific). For the following assays, cells were seeded in triplicates.

### 2.2. Drug preparation

HQ (Cat no: 259343-5G) was purchased from Sigma-Aldrich and dissolved in DMSO (Cat no: P60-36720100, Pan Biotechnology) to get 10 mM concentration. Prepared HQ solution was aliquoted and stored at −20 °C until use. HQ was then diluted with culture medium to obtain concentrations ranging from 0.05 mM to 0.8 mM for the experiments.

### 2.3. Determination of IC50 values

CellTiter 96^®^ Non-Radioactive Cell Proliferation Assay kit (Cat no: G400, Promega) was utilized to determine the half minimal inhibitory concentration (IC50) values for MCF-7 and SKOV-3 cells. In a 96-well plate (Thermofisher Scientific), 1 × 10^4^ cells were seeded in a 0.1 mL medium for 24-h treatments. On the other hand, for 48-h treatments, 0.5 × 10^4^ cells were seeded in order to avoid cells becoming overconfluent considering the longer incubation time. Cells were incubated 24 h, and then culture mediums were replaced with HQ-containing mediums with 0.05 mM, 0.1 mM, 0.2 mM, 0.4 mM, 0.6 mM, and 0.8 mM concentrations. For negative and positive controls, culture medium without HQ and culture medium with 10% DMSO were used, respectively. After the incubation, the MTT assay was performed according to the manufacturer’s protocol. For IC50 values calculation “drc” package, and for data visualization “ggplot2” package as described elsewhere (Sibai et al., 2019) in R language (4.3.2) were utilized.

### 2.4. Colony formation assay (CFA)

CFA was performed to assess HQ’s effect on the colony forming ability of the analyzed cancer cells. In a 96-well plate, 0.1 mL of medium containing 2 × 10^3^ cells were seeded. Experimental group was comprised 0.1 mM, 0.2 mM, and 0.4 mM HQ in culture medium, and as a positive control HQ-free fresh culture medium was used. When the control group reached 80% confluency, which took 5 days for both cells, the assay was finalized as follows (Demircan et al., 2021). First, the cells were fixed using 0.15 mL 100% methanol (Cat no: 1.06009.2511, Merck) for 20 min at room temperature (RT). Then, the colonies were stained with 0.1 mL 0.2% Crystal Violet Solution (Cat no: C077, Sigma) for 15 min. Subsequently, to eliminate the background staining, the formed colonies were washed twice with 0.1 mL ddH_2_O. The plate was left overnight to dry. As a last step, microphotographs were taken at 4X magnification and analyzed utilizing the “ColonyCounter” plugin in ImageJ Software (1.8.0).

### 2.5. Wound healing assay (WHA)

To test the migration ability of the cells upon HQ treatment, WHA was employed. 1 × 10^5^ cells were seeded in a 24-well plate (Thermofisher Scientific) in a 1 mL medium. The next day, mediums were replaced with 0.5 mL culture medium containing 0.2 mM HQ or culture medium without HQ for experimental and control groups, respectively. After 24 h treatment, straight wounds were created using p200 pipette tips, and the microphotographs were taken on the 0^th^, 6^th^, and 24^th^ h after the scratch. All images were analyzed with the “MRI Wound Healing” plugin of ImageJ software. The wound area calculated from the images taken at the beginning (0^th^ h) was considered 100, and the percentage of the cells closing the wound areas at the 6^th^ and 24^th^ h was found by the ratio of the wound area at these time points to the 0^th^ h.

### 2.6. Soft agar assay

In order to evaluate the impact of HQ on tumorigenicity, 3-D soft agar model was implemented by following a previously established protocol (Du et al., 2017). To prepare the bottom layer, 0.5% agar solution (Cat no: A1296-500G, Sigma) and culture medium were mixed at 1:9 v/v. Each well of the 12-well plate was filled with 0.8 mL of the agar-medium mixture (Thermofisher Scientific) and each well was then incubated for 30 min at RT. Cell number was adjusted to 12 × 10^3^ for each well. Then, 0.94 mL cell suspension was mixed with 0.06 mL of 0.5% agar solution, 0.8 mL of the mixture was added on the top of each bottom layer to form the upper layer, and incubated for 30 min at RT. Following the incubation, 0.8 mL of culture medium with or without 0.2 mM HQ was added to the top of the upper layer as a feeder layer. All cultures were incubated till the control cells formed significant spheres, which was 21 days for MCF-7, and 35 days for SKOV-3 cells. Feeder layers were changed twice a week. Following the incubations, microphotographs were taken at 4X magnification, and spheroid size and numbers were measured with ImageJ software.

### 2.7. Annexin-V assay

To link the anticarcinogenic effect of HQ with cell death rate, apoptosis assay using Alexa Fluor^®^488 Annexin V/Dead Cell Apoptosis kit (Cat no: V13242, Thermofisher Scientific) was conducted to detect apoptotic cell numbers as described earlier (Hacıoğlu et al., 2020) in HQ-treated and control groups. 1 × 10^5^ cells were seeded in a 12-well plate in a 1 mL medium. After 24 h, the mediums were changed with 1 mL culture medium with or without 0.2 mM HQ to form experimental and control groups. After 24 h incubation, cells were washed with 1 mL cold PBS (Cat no: 003002, Thermofisher Scientific). Washed cells were pelleted at 1500 rpm for 5 min and resuspended in 0.1 mL 1X Annexin-binding buffer. Then, 5 μL Alexa Fluor^®^488 dye and 1 μL 100 μg/mL of PI working solution were added to cell suspensions, and cells were incubated for 15 min at RT, protected from light. Subsequently, 0.4 mL 1X Annexin-binding buffer was added to each tube, and the samples were analyzed by flow cytometry (BD Accuri™ C6 Plus).

### 2.8. Carboxyfluorescein succinimidyl ester (CFSE) CellTrace assay

CFSE assay (Cat no: C34554, Thermofisher Scientific) was employed to assess cellular proliferation rate upon HQ treatment as described before (Yazdi et al., 2018). In a 24-well plate, 1 × 10^5^ cells were seeded in a 1 mL medium. After 24 h of incubation, 0.5 μL CFSE was mixed with 0.5 mL serum-free medium, and cells were incubated with this mixture for 20 min at 37 °C. After the incubation, cells were washed with 0.5 mL complete medium twice, and 0.5 mL culture medium containing 0.2 mM HQ or culture medium without HQ was used to replace the medium before a 24-h incubation. When the treatment was completed, cells were harvested, washed with 1 mL PBS, and then resuspended in 0.5 mL fresh PBS. The proliferation analysis of cells was performed using BD Accuri™ C6 Plus at 488 nm.

### 2.9. Protein extraction and sample preparation

To explore the HQ’s effect at the molecular level, proteomics was performed. In a 6-well plate (Thermofisher Scientific), 3 × 10^5^ cells were seeded in a 2 mL medium, and a 24-h incubation was followed for the cell attachment. Following incubation, 0.2 mM HQ in 1 mL culture medium or culture medium without HQ was added to cells, and the cells incubated for 24 h. Then, cells were harvested, washed with 1 mL PBS, and pelleted. The pellets were lysed and homogenized with sonication in UPX solution (Expedion) containing protease inhibitor cocktail (Thermo Scientific). Filter aided sample preparation (FASP) method was performed to obtain tryptic peptides. Briefly, the cell lysates were reduced with dithiothreitol (DTT) and alkylated with iodoacetamide (IAA), respectively. Samples were centrifuged at 14,000 × g for 10 min, and the supernatant was collected. Protein concentration was determined by Bradford Protein Assay prior to the trypsinization step. Trypsin (Promega) was then added at 1:100 (w/w), and digestion was carried out for 18 h at 37 °C. Peptide concentrations were measured by Quantitative Fluorometric Peptide Assay (Pierce) prior to LC-MS/MS analysis. Before LC-MS analysis, the final peptide concentration was adjusted to 200 ug/mL with %0.1 formic acid.

### 2.10. LC-MS/MS analysis and data processing

LC-MS/MS analysis was performed as in our previous studies (Demircan et al., 2017; Sibai et al., 2020). Briefly, during sample preparation with the FASP (Filter aided sample preparation protocol) kit, the extracted proteins are filtered through a 30 kDa cut-off spin column which effectively eliminates any interfering compounds, and at the same time, endogenous peptides. The resulting intact protein mixture is then incubated to generate tryptic peptides. A 200 ng tryptic peptide mixture was analyzed by nano-LC-MS/MS system (Acquity UPLC M-Class and SYNAPT G2-Si HDMS; Waters. Milford, MA, USA). Peptide mixtures are loaded on the trap column (Symmetry C18 5 mm, 180 mm i. d. 20 mm) and then separated by analytic column (CSH C18, 1.7 mm, 75 mm i. d. 250 mm) with a linear 2h gradient (4%–40% Acetonitrile 0.1% (v/v) FA, 0.300 mL/min flow rate). A hundred fmol/uL Glu-1-fibrinopeptide-B was used as lock mass reference at 0.500 mL/min flow rate with 60 s intervals. A data-independent acquisition mode called SONAR (Juvvadi et al., 2018) was used for MS data acquisition with a 24 Da quadrupole transmission width. Positive ionization mode was used at 50–2000 m/z in the full scan mode.

Progenesis QI for proteomics (v.4.0, Waters) was used for the quantitative analysis of peptide features and protein identification. Processing parameters were 60 counts for the low energy threshold and 10 counts for the elevated energy threshold. Total ion intensity was used for normalization between samples. Expressional changes and p values were calculated with the statistical package included in Progenesis QI for proteomics, and protein normalization was performed according to the relative quantitation using nonconflicting peptides. The resulting data set was filtered by ANOVA p-value 0.01, and only proteins with a differential expression level between the two conditions greater than or equal to 1.2-fold change (FC) were considered.

### 2.11. Validation of proteomics data

Proteomics data were validated by reverse transcription quantitative polymerase chain reaction (RT-qPCR) method using gene-specific primers presented in Supplementary File S1. Five high- or low-abundant proteins were selected for RT-qPCR validation for MCF-7 and SKOV-3 cells. RNA isolation was carried out *via* NucleoSpin RNA Plus (Cat no: 740984.50, Macherey-Nagel) following the manufacturer’s instructions. RNA integrity and concentration were examined using 1% Agarose gel electrophoresis and Nanophotometer^®^ N50 (IMPLEN), respectively. RT-qPCR was conducted using GoTaq^®^ 1-Step RT-qPCR System (Cat no: A6020, Promega) according to producer’s protocol.

### 2.12. Protein–protein interactions networks

STRINGdb v11.0 was used to construct and visualize the protein–protein interaction (PPI) networks (Szklarczyk et al., 2019). To analyze the clustering and interaction of differentially abundant (DA) proteins, high- and low-abundant proteins in HQ treated MCF-7 and SKOV-3 cells were queried. DE proteins enriching the top 5 biological process (BP) terms were labeled with different colors.

### 2.13. Visualization of proteomics data

DA proteins (p-value < 0.01 and |FC| > 1.2) were displayed on a volcano plot using the “enhanced volcano” R package as implemented before (Demircan et al., 2021). The top 5 high- and low-abundant proteins were highlighted in the plots. Same lists of DA proteins were subjected to gene ontology (GO) and Kyoto Encyclopedia of Genes and Genomes (KEGG) enrichment analyses using “clusterProfiler” package (Yu et al., 2012) in R (version: 4.1.0). ReactomePA package (Yu and He, 2016) was used to enrich the DA proteins in Reactome pathways. Benjamini & Hochberg (BH) was applied in enrichment tests as the adjusted p-value method, and cutoffs of adjusted p-value and q-value were set to 0.05. The “org.Hs.eg.db” (human) database was selected as the background gene list. The top enriched pathways and processes were plotted using “ggplot2” and “ggpubr” R packages.

### 2.14. Statistical analysis

Statistical analysis of the generated data and visualization were performed using the R language (version 4.3.2). For the evaluation of data distribution, the Shapiro-Wilk test was utilized. For normally distributed data, one-way analysis of variance (ANOVA) followed by Tukey’s post hoc test was applied. Welch’s t-test was used in the pairwise comparisons. All experiments were performed in triplicate, each being repeated at least three times. Data were presented in the figures as mean ± standard deviation (SD). Statistical significance was set to p-value < 0.05.

## 3. Results

### 3.1. HQ exhibited a cytotoxic effect on SKOV-3 and MCF-7 cells

**Figure 1:**
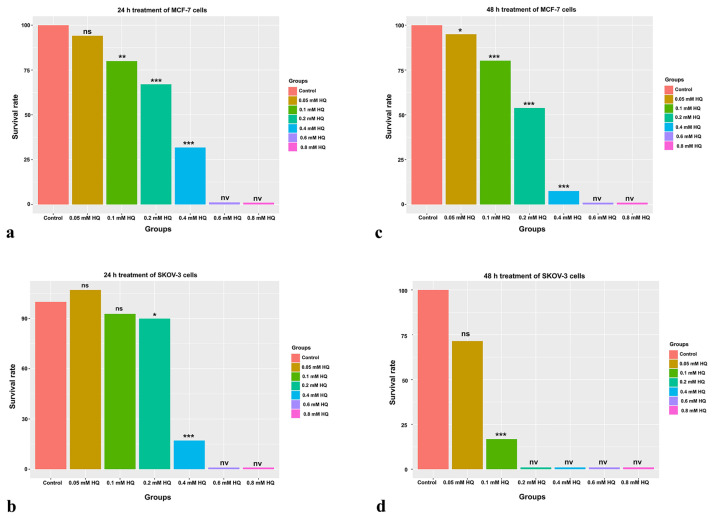
Determination of IC50 values. MTT was performed to determine IC50 values and assess the cytotoxicity of HQ on MCF-7 and SKOV-3 cells. a) Survival rate of MCF-7 cells upon HQ treatment for 24 h. The survival rate for: Control group: 100 ± 7.1, 0.05 mM HQ group 94 ± 5.5, 0.1 mM HQ group 80 ± 1.6, 0.2 mM HQ group 67 ± 0.4, 0.4 mM HQ group 32 ± 2.0, 0.6 mM HQ group 1 ± 1.1, 0.8 mM HQ group 1 ± 0.1 b) The survival rate of MCF-7 cells upon HQ treatment for 48 h. The survival rate for: Control group 100 ± 2.0, 0.05 mM HQ group 95 ± 4.5, 0.1 mM HQ group 80 ± 4.5, 0.2 mM HQ group 54 ± 6.0, 0.4 mM HQ group 8 ± 0.4, 0.6 mM HQ group 1 ± 0.2, 0.8 mM HQ group 1 ± 0.1 c) Relative viability of HQ-treated SKOV-3 cells throughout 24 h. The survival rate for: Control cells 100 ± 5.0, 0.05 mM HQ group 107 ± 3.4, 0.1 mM HQ group 93 ± 1.0, 0.2 mM HQ group 90 ± 2.7, 0.4 mM HQ group 17 ± 1.4, 0.6 mM HQ group 1 ± 0.3, 0.8 mM HQ group 1 ± 0.2 d) The effect of 48-h HQ treatment on SKOV-3 cells. The survival rate for: Control group 100 ± 3.4, 0.05 mM HQ group 71 ± 4.1, 0.1 mM HQ group 17 ± 2.2, 0.2 mM HQ group 1 ± 0.1, 0.4 mM HQ group 1 ± 0.1, 0.6 mM HQ group 1 ± 0.1, 0.8 mM HQ group 1 ± 0.1 *p-value < 0.05, **p-value < 0.01, ***p-value < 0.001, ns; nonsignificant, nv; nonviable. HQ; hydroquinidine.

A wide range of HQ concentration (0.05 mM, 0.1 mM, 0.2 mM, 0.4 mM, 0.6 mM, and 0.8 mM) was tested on MCF-7 and SKOV-3 cells to assess its cytotoxicity on breast and ovarian cancer cells and determine the half maximal inhibitory concentrations (IC50). HQ decreased the cell viability for both cancer cell in a time- and dose-dependent manner (Figure 1) (Supplementary Table S1). For 24-h treatment groups IC50 values were found as 0.31 mM for MCF-7 cells and 0.28 mM for SKOV-3 cells. As exposure to HQ prolongs, the cytotoxicity enhanced and IC50 values were decreased and calculated as 0.21 mM for MCF-7 cells and 0.065 mM for SKOV-3 cells.

### 3.2. HQ significantly limited the cancer cell characteristics of MCF-7 cells

**Figure 2:**
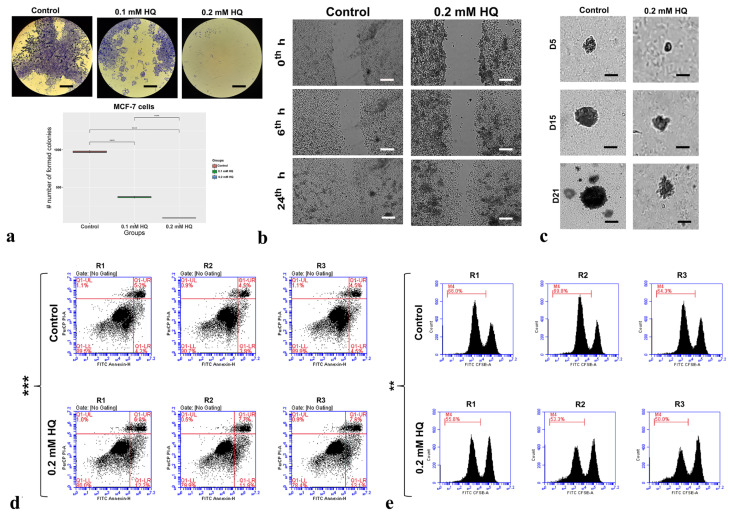
HQ’s anticarcinogenic activity on MCF-7 cells. a) CFA was utilized to evaluate the impact of HQ on clonogenicity. Representative images of control or HQ-treated MCF-7 groups. b) The migration capacity of cells was detected utilizing WHA. Representative images of MCF-7 cells at 0^th^-, 6^th^-, and 24^th^-h time points. c) The evaluation of HQ’s effect on MCF-7 spheroids was conducted by implementing a soft agar assay. Representative images of MCF-7 spheroids in control and HQ treatment groups. d) Annexin-V method was adopted to determine cellular apoptosis. Flow charts of MCF-7 cells e) CFSE was used to measure the proliferation rate. CFSE histograms of MCF-7 cells. **p-value < 0.01, ***p-value < 0.001. HQ; hydroquinidine. R; replica. D; day. Photomicrographs were taken at a magnification of 4X. Scale bar: 50 μm

As a next step, we examined the HQ effect on the colony forming ability, migration capacity, tumorigenicity, apoptosis, and proliferation rate. The obtained IC50 value, 0.2 mM, was used as HQ concentration for the experimental group to compare with untreated cells. It was noted that 0.2 mM HQ treatment impaired colony forming ability of the cells by 10-fold (p-value < 0.001) (Figure 2a). A significant decrease (3-fold, p-value < 0.001) in the number of formed colonies was also observed for 0.1 mM HQ treatment (Figure 2a). Moreover, migration capacity was remarkably reduced in HQ-treated cells by 1.6-fold (Figure 2b). The anticarcinogenic activity of HQ was further confirmed by tumorigenicity assay (Figure 2c). Drug treatment significantly reduced the number (3.14-fold) and size (2-fold) of the formed spheroids (p-value < 0.001). Then, the effect of HQ on proliferation and apoptosis was inspected. Remarkably, HQ exerted a proapoptotic and antiproliferative effect on MCF-7 cells (Figure 2d, 2e). Apoptotic cell number in 0.2 mM HQ-treated group was 2.2-fold higher than the control group (p-value < 0.001) (Figure 2d). Furthermore, a significant decrease (1.26-fold, p-value < 0.01) in cell division rate was detected in drug-administrated MCF-7 cells (Figure 2e). Altogether, our data indicated the antigrowth impact of HQ on MCF-7 cells.

### 3.3. HQ displayed a significant antineoplastic effect on SKOV-3 cells

**Figure 3:**
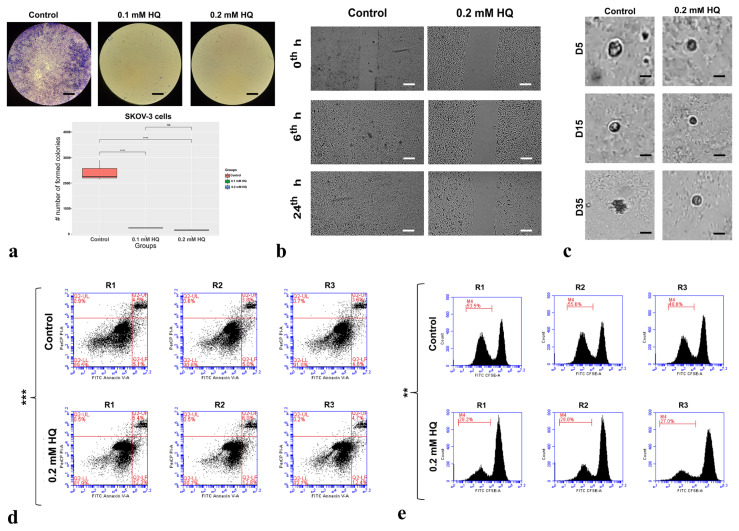
HQ’s antineoplastic effects on SKOV-3 cells. a) The colony forming ability of the cells was assessed using CFA. Representative images of SKOV-3 colonies. b) WHA was employed to evaluate the migration capacity of the cells. Representative images of SKOV-3 cells upon creating a scratch at 0^th^-, 6^th^-, and 24^th^-h time points. c) Soft agar assay was conducted to observe HQ’s impact on SKOV-3 spheroids. Representative images of SKOV-3 cells from control and HQ-treatment groups. d) Apoptosis rate was determined utilizing Annexin-V assay. Apoptosis rate of SKOV-3 cells. e) Proliferation rate was evaluated via CFSE assay. The proliferation rate of SKOV-3 cells. **p-value < 0.01, ***p-value < 0.001. HQ; hydroquinidine. R; replica. D; day. Photomicrographs were taken at a magnification of 4X. Scale bar: 50 μm

For treated SKOV-3 cells, a similar result as in MCF-7 cells was observed (Figure 3). The ability of SKOV-3 cells to form colonies was reduced by 10- and 14-fold by 0.1 mM and 0.2 mM HQ, respectively (p-value < 0.001) (Figure 3a). Strikingly, 0.2 mM HQ treatment significantly decreased the migration capacity of SKOV-3 cells by 19-fold (p-value < 0.001) (Figure 3b). On top of the considerable decrease in colony-forming ability and migration capacity, HQ also significantly reduced SKOV-3 tumorigenicity (Figure 3c). Upon incubation with the drug, the level of decline was detected as 3.2-fold and 2-fold for the number and size of formed spheroids, respectively (p-value < 0.001). Next, we sought the HQ activity on cell death and proliferation. In HQ-treated group, a 2.03-fold increase (p-value < 0.001) in apoptosis and a 1.25-fold decrease (p-value < 0.01) in proliferation rate was detected (Figures 3d, 3e). Altogether, HQ suppresses several cancer hallmarks for SKOV-3 cells.

### 3.4. HQ altered the protein expression profile of MCF-7 and SKOV-3 cells

In order to uncover the molecular mechanisms of the HQ’s impact, proteomic analysis of 0.2 mM HQ-treated MCF-7 and SKOV-3 samples with their nontreated controls was conducted. Among the identified proteins, without any p-value and FC cutoffs, 1065 of them were detected between HQ-treated and control MCF-7 cells, and 199 were found as significantly (p-value < 0.05, |FC| >1.2) high-or low-abundant (45 and 154, respectively). For SKOV-3 cells, out of 1074 proteins, 45 proteins were significantly high-abundant (p-value < 0.05, FC > 1.2), and 43 proteins were significantly low-abundant (p-value < 0.05, FC < 1.2) in the HQ treatment group compared to the control one. Selected 5 high- or low-abundant proteins were validated by RT-qPCR for both MCF-7 and SKOV-3 cells (Supplementary File S1). After the drug treatment, a similar altered gene expression trend at the mRNA level confirmed proteomics results.

**Figure 4:**
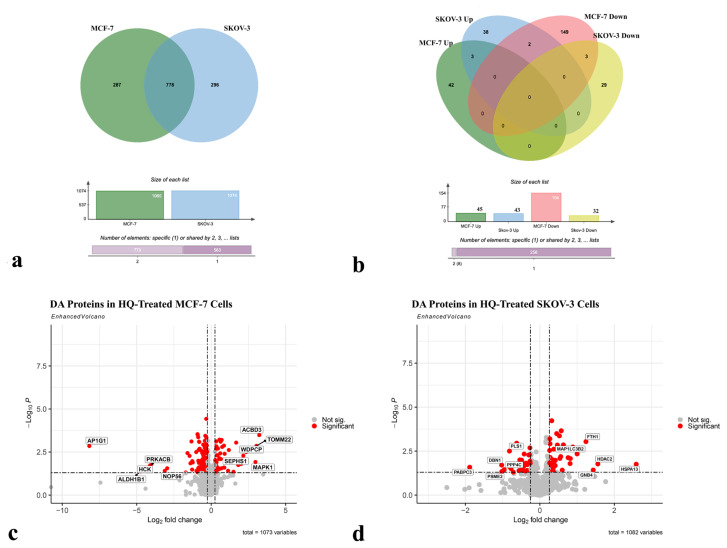
Comparison of commonality and significance of all identified and DA proteins. a) Venn diagram of identified proteins in MCF-7 and SKOV-3 cells, b) Venn diagram of DA proteins up-and down-regulated in MCF-7 and SKOV-3 cells, Volcano plots of identified proteins c) in MCF-7 and d) SKOV-3 cells. The red color shows significant DA proteins (p < 0.05 and FC > 1.2), and the grey color indicates nonsignificant DA proteins.

Seven hundred and seventy eight shared proteins between MCF-7 and SKOV-3 proteome were detected (Figure 4a, Table S2). Exploration of the DA proteins commonality between MCF-7 and SKOV-3 resulted in three commonly low-abundant (EIF4G1, PLS1, and MT-CO2) and three commonly high-abundant proteins (RAB2A, OAT, and SQSTM1) (Figure 4b, Table S3). DDX6 and H2AZ1 proteins were significantly found to be high-abundant in HQ treated SKOV-3 samples and low-abundant in HQ-treated MCF-7 samples. The cancer type-specific differential expression profile due to HQ treatment was noteworthy.

All identified proteins, whose top 5 proteins with the highest FC value were highlighted, were illustrated on a volcano plot (Figures 4c–4d). ACBD3, TOMM22, WDPCP, MAPK1, and SEPHS1 were top high-abundant, whereas P1G1, PRKACB, HCK, NOP56, and ALDH1B1 were top low-abundant proteins in HQ-treated MCF-7 cells (Figure 4c). On the other hand, in HQ-treated SKOV-3 cells, FTH1, HDAC2, HSPA13, GNB4, and MAP1LC3B2 were found to be top high-abundant, and PLS1, PPP4C, DBN1, PSME2, and PABPC3 were top low-abundant proteins (Figure 4d).

Among DA proteins, several of them were randomly evaluated to check whether their roles in cancer progression or inhibition were identified by previous studies. As shown in Table S4, 30 DA proteins associated with pro or anticarcinogenesis were listed. The whole list of DA proteins that potentially include more cancer-related proteins than in Table S4 can be found in Table S3.

### 3.5. Enriched BP terms in GO analysis and gene set enrichment analysis (GSEA) confirms the observed toxicity of HQ

**Figure 5:**
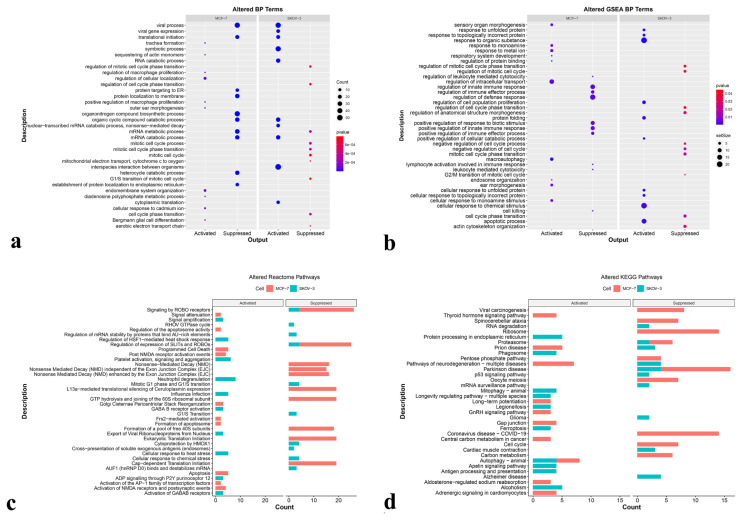
GO and GSEA BP terms, and KEGG and Reactome pathways enriched by DA proteins. a) Enriched top 10 GO analyses associated BP’s by DA proteins, b) Enriched top 10 GSEA associated BP’s by DA proteins, c) Enriched top 10 KEGG pathways by DA proteins, d) Enriched top 10 Reactome pathways by DA proteins. For a) and b), the dot size is proportional to the number of genes, and color indicates the significance. For c) and d), bar size is proportional to the number of genes, and color indicates the cell type.

We then sought the enriched BP terms by significant DA proteins through GO analysis (Figure 5a) and GSEA (Figure 5b). Activated terms specified the terms enriched by significantly upregulated proteins, and suppressed terms indicated those enriched by significantly downregulated proteins in HQ-treated cells. For MCF-7 cells, 60 and 82 BP terms were identified as activated and suppressed, respectively, and the top three terms in the activated BP list were “trachea formation”, “regulation of cellular localization”, and “outer ear morphogenesis”. Top BPs in the suppressed list were detected as “viral process”, “mRNA catabolic process”, and “translational initiation”.

On the other hand, 87 activated and 61 suppressed BP terms were described for SKOV-3 cells. The top three activated BPs were “protein folding”, “viral life cycle”, and “chaperone cofactor-dependent protein refolding”. Whereas “mitochondrial electron transport, cytochrome c to oxygen”, “aerobic electron transport chain”, and “mitotic cell cycle phase transition” were defined as top BPs among the suppressed terms. Enriched BP terms were listed in Table S5. Strikingly, several suppressed BPs in HQ treated SKOV-3 group were related to cell cycle, such as “cell cycle phase transition”, “G1/S transition of mitotic cell cycle”, “mitotic cell cycle”, “mitotic cell cycle transition”, “regulation of cell cycle phase transition”.

GSEA uses a different algorithm to enrich the ontology terms; therefore, it may provide valuable information not presented in GO analysis. One hundred and fifty-three BPs (108 activated, 45 suppressed) were enriched by significant DA proteins in the MCF-7 proteome. Activated BPs with the lowest p-value were found to be “regulation of protein binding”, “respiratory system development”, and “regulation of intracellular transport”, while the top three suppressed terms were determined as “positive regulation of immune effector process”, “regulation of leukocyte mediated immunity”, and “positive regulation of leukocyte mediated immunity”. On the contrary, for the SKOV-3 proteome, significant DA proteins enriched 95 GSEA BPs, of which 80 were activated and 15 were suppressed. In HQ treated SKOV-3 cells, upregulated proteins enriching the top three terms were identified as “apoptotic process”, “response to unfolded protein”, and “cellular response to unfolded protein”, whereas “actin cytoskeleton organization”, “cell cycle phase transition”, and “mitotic cell cycle phase transition” were the top suppressed terms upon HQ treatment. The lists of enriched GSEA BP terms for MCF-7 and SKOV-3 cells were provided in Table S6.

### 3.6. Altered KEGG and Reactome pathways were in parallel with BP terms

Reactome (Figure 5c) and KEGG (Figure 5d) pathway analyses were performed to get more insights into HQ antineoplastic activity. Significantly upregulated proteins in MCF-7 cells treated with HQ yielded 76 Reactome pathways, and the top three of those were “Golgi Cisternae Pericentriolar Stack Reorganization”, “post NMDA receptor activation events”, and “activation of NMDA receptors and postsynaptic events”. Although not being represented in the top three pathways, activation of “apoptosis”, “programmed cell death”, “formation of apoptosome”, “regulation of the apoptosome activity”, “apoptotic factor-mediated response”, “apoptotic cleavage of cellular proteins”, and “apoptotic execution phase”, Reactome pathways implied the increased cell death activity upon HQ treatment. While out of 117 suppressed pathways in MCF-7 cells, “L13a-mediated translational silencing of Ceruloplasmin expression”, “GTP hydrolysis and joining of the 60S ribosomal subunit”, and “regulation of expression of SLITs and ROBOs” were the most significant pathways for p-value. However, the suppression of proliferation related pathways, including “DNA replication preinitiation”, “G1/S transition”, “mitotic G1 phase and G1/S transition”, “M phase”, “G2/M transition”, and “mitotic prophase” underlined the antiproliferative activity of HQ.

For SKOV-3 cells, 162 Reactome pathways were activated, and 146 pathways were suppressed following the HQ treatment. Among the activated ones, “regulation of HSF1-mediated heat shock response”, “cellular response to heat stress”, and “ADP signalling through P2Y purinoceptor 12”, and out of suppressed ones, “cytoprotection by HMOX1”, “AUF1 (hnRNP D0) binds and destabilizes mRNA”, and “mitotic G1 phase and G1/S transition” were the top three pathways. In addition, while several signaling and autophagy-related pathways were detected as activated, cell-cycle and DNA damage response pathways were suppressed for HQ treated SKOV-3 cells. Enriched Reactome pathways are listed in Table S7.

Since the pathway databases vary in the number of pathways, the number of proteins per pathway, and the types of subcategories they provide, consideration of more than one database may increase the quality of analysis. Thus, we conducted KEGG pathway analysis (Figure 5d) alongside the Reactome pathway enrichment. Significantly upregulated proteins enriched 96 KEGG pathways in HQ-treated MCF-7 cells. Although “gap junction”, “aldosterone-regulated sodium reabsorption”, and “pathways of neurodegeneration-multiple diseases” were the top three pathways, enrichment of “autophagy-animal” pathway was notable. Out of 24 pathways enriched by significantly downregulated proteins in HQ-treated MCF-7 cells, “ribosome”, “coronavirus disease-COVID-19”, and “viral carcinogenesis” were the top three pathways. Besides, enrichment of proliferation-related pathways, including “cell cycle”, “oocyte meiosis”, and “DNA replication” was remarkable.

DA proteins in SKOV-3 proteome activated 28 KEGG pathways in which the top three were as follows: “Mitophagy-animal”, “antigen processing and presentation”, and “ferroptosis”. Identification of several other KEGG pathways related to increased intracellular stress and cell death, such as “autophagy-animal”, “phagosome”, and “necroptosis” was striking. Moreover, 22 KEGG pathways were suppressed upon HQ treatment in SKOV-3 cells. Pathways with the lowest p-value were detected as “cardiac muscle contraction”, “Parkinson disease”, and “proteasome”. A full list of identified KEGG pathways for each condition is demonstrated in Table S8.

### 3.7 Investigation of DA proteins interactions

**Figure 6:**
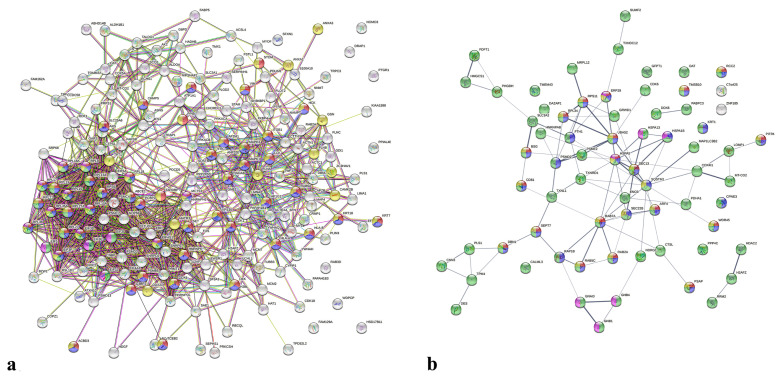
Multicenter PPI network of DA proteins in MCF-7 and SKOV-3 cells. PPI networks were formed by implementing STRING analysis. a) DA proteins in MCF-7 generating PPI enrichment map (p-value < 1.0e–16). b) PPI network of DA proteins in SKOV-3 samples (p-value < 0.000599). Colors signify the most enriched BP terms in these networks as follows: Red; “symbiotic process”, blue; “viral process”, green; “translational initiation”, yellow; “interspecies interaction between organisms”, purple; “cytoplasmic translation” for MCF-7 cells. For SKOV-3 cells red; “cellular protein localization”, blue; “cellular localization”, green; “cellular process”, yellow; “protein localization”, purple; “protein folding”.

Next, we analyzed the interaction of DA proteins through the implementation of the STRING database. PPI of MCF-7 DA proteins (Figure 6a) resulted in 194 nodes and 1082 edges, and the PPI enrichment p-value was <1.0e–16. For SKOV-3 DA proteins (Figure 6b), 69 nodes and 91 edges were detected with a PPI enrichment p-value of 0.000599. HSPA5, RAB7A, and SQSTM1 were identified as the core proteins in the PPI network with the highest node degree for SKOV-3 cells. It was noted that almost all DA proteins were connected and interacted. The highest node degree of EFTUD2, RPS5, and EIF3I positioned these proteins as the top 3 core proteins in the PPI network of MCF-7 cells. A highly associated PPI map, particularly clustered around the proteins enriching translation-related pathways, was striking.

## 4. Discussion

Many females are struggling with breast and ovarian cancers worldwide. Since these two types of cancer are highly metastatic and the treatment options are limited, they are among the leading causes of women’s death globally. Despite the increased understanding of the molecular basis of these two cancer types and the advancement of medical applications, current therapies are still not efficient and convenient for many patients. Therefore, the discovery of innovative therapeutic approaches is highly required. Recent studies described ion channel blockers as promising anticancer compounds. In this respect, we interrogated the growth limiting the potential of HQ, an ion channel blocker, in breast and ovarian cancers.

To our best knowledge, HQ anticancer activity has not been reported so far. In the literature, there is an anecdotal study of HQ utilization in cancer treatment to overcome drug resistance (Chauffert et al., 1997). This study tested HQ on various multiple-drug-resistant (MDR) cancers including leukemia, neuroblastoma, lymphoma, lung, and ovarian carcinoma (Chauffert et al., 1997). On these cancer cells, doxorubicin (DXR) anticarcinogenic activity was compared with its combination with HQ, quinine, or verapamil. The data indicated that HQ significantly increased DXR’s anticarcinogenic effect by increasing cytoplasmic DXR concentration. This finding was further supported by in vivo results where the intraperitoneal injection of DXR-HQ combination to rats elevated intracytoplasmic DXR levels (Chauffert et al., 1997).

Here, we first tested HQ’s antigrowth potential on MCF-7 and SKOV-3 cells and observed substantial toxicity in these cells (Figures 2,3). Due to the limited research on HQ’s anticarcinogenic effect, we searched the literature regarding chemically similar compounds. In line with our data, in a recent study, semisynthetic quinine derivatives quinoline and quinuclidine exhibited a noteworthy cytotoxic impact on breast and ovarian cancer cells (Akhtar et al., 2020). Moreover, the anticancer activity of cinchona bark compounds on cervical and lung adenocarcinomas was previously highlighted (Li et al., 2017; Qi et al., 2017, p. 6). Furthermore, cinchona bark compounds suppressed tumor growth in mice (Qi et al., 2019). Another study uncovered that growth inhibition by quinine treatment in lung adenocarcinoma and cervical cancer cell lines is due to the impaired activity of TRAF6, a key regulator of Akt signaling (Liu et al., 2016). Considering the similarity in the chemical structure of HQ and quinine derivatives, an overlap of interactome among cinchona bark compounds is plausible, and the common target of these compounds may link the observed anticarcinogenic activities in our findings with previous studies.

As a next step, we investigated the HQ’s effect on migration. Our results demonstrated that HQ exhibited a significant antimigratory activity on SKOV-3 cells and a substantial decrease in MCF-7 cells. In MDA-MB-435S melanoma cells blocking of KCNH2 (HERG) potassium channel via E4301 or cisapride attenuated both proliferation and migration (Afrasiabi et al., 2010) while activation of KCNH2 using PD118057 exhibited an opposite effect. In endometrial cancer cells, inhibition of potassium channels either with glibenclamide or 4-aminoprydine restricted proliferation and migration (Erdem Kış et al., 2022). In breast cancer cells, FDA-approved potassium blocker amiodarone decreased the migration capacity in vitro, and metastasis and tumor growth in vivo through Cadherin-11 (Payne et al., 2022). In line with our data, the knockdown of KCNH3 potassium channel in ovarian cancer cells, SKOV-3 and COC-1, significantly reduced proliferation and migration while increasing apoptosis (Li et al., 2020). Moreover, downregulation of HERG suppressed tumor growth in vivo and migration and invasion of SKOV-3 cells in vitro (Zhi et al., 2019). On the contrary, tetrodotoxin (TTX), a well-characterized sodium channel blocker, had no significant effect on the migration potential of MDA-MB-231, MCF-7, and MDA-MB-468 cells (Roger et al., 2003). Concordantly, siRNA mediated Nav1.5 inhibition did not affect the MCF-7 migration capacity (Mohammed et al., 2015). Therefore, as an ion channel blocker, we concluded that HQ displayed a similar antimigrative effect on SKOV-3 cells but not on MCF-7 cells, as previously shown by earlier reports.

Our findings revealed that HQ induces apoptosis and suppresses proliferation in breast and ovarian cancer cell lines (Figures 2d–2e, Figures 3d–3e). In accordance with our findings, it was demonstrated that quinidine blocks the cell cycle via G0/G1 arrest in MCF-7 (Melkoumian et al., 2002; Solh et al., 2008). In another study, decreased proliferation rate in quinidine-treated MCF-7 cells was linked to the elevated level of CDKN1A, suppressed retinoic acid signaling, and downregulation of cyclin D1 (Q. Zhou et al., 2002). Additionally, quinidine decreased liver and cervical carcinoma cell proliferation by promoting G0/G1 arrest and apoptosis (El-Mesery et al., 2021). Likewise, quinoline and quinuclidine increased G0/G1 arrest and apoptosis in MCF-7 cells and the Ehrlich ascites carcinoma murine model (Akhtar et al., 2020). G0/G1 arrest and increased apoptosis were also reported by quinine activity in choriocarcinoma (Nilkaeo et al., 2006). Moreover, treatment of cinchona alkaloids led to a significant induction of apoptosis in HeLa cells via the Akt and TAK1 inactivation and BAX/BCL2 upregulation (Qi et al., 2019). The same study uncovered increased apoptosis in the syngeneic mouse cancer model (Qi et al., 2019). Inhibition of ion channels by E3Ab or lidocaine in ovarian, uterus, and breast carcinoma cells significantly decreased the proliferation rate (Gao et al., 2019). In the Caov-3 xenograft model, E3Ab or lidocaine treatment suppressed tumor growth characterized by less mitotic activity (Gao et al., 2019). Altogether, our study confirmed the previous findings on the impact of ion channel blocking or treatment with quinine derivatives to stimulate apoptosis and limit proliferation rate.

As a complementary analysis, the molecular basis of observed HQ’s antineoplastic effect was investigated by proteomic analysis. Most of the identified DA proteins between control and HQ-treated MCF-7 and SKOV-3 cells are associated with cancer-related processes (Figures 5c–5d, Table S4) and confirm previously published results. For the HQ-treated MCF-7 cells, Reactome and KEGG proliferation pathways were enriched primarily by CDK1, PSMB5, PSMC2, MCM2, MCM7, YWHAH, YWHAQ, and YWHAB proteins that were found to be significantly low-abundant (Table S7, S8).

Inhibition of *CDK1* suppressed the endometrial and breast cancer cell viability and colony-forming capacity (Xia et al., 2014). *CDK1* inhibition stimulated G2/M arrest and apoptosis in multiple cancer cell lines (Xia et al., 2014; Ying et al., 2021) and decreased tumor growth in vivo in the mouse xenograft model (Ying et al., 2021). Several proteasome subunit genes (PSM) are significantly low-abundant in HQ-treated MCF-7 and SKOV-3 cells. *PSMB5* inhibition reduced formed colonies and migration in MDA-MB-231 cells and suppressed tumor growth at early stages in in vivo (Wang et al., 2017). Silencing of another proteasome subunit, proteasome 26S subunit ATPase (PSMC2), remarkably reduced hepatocellular (Liu et al., 2021), prostate (Liu et al., 2021), breast (Wang et al., 2021), and gastric (Liu et al., 2022) cancers viability, migration and invasion capacity, while promoting cell-cycle arrest and consequently apoptosis in these cell lines. The same studies reported tumor growth limiting activity upon *PSMC2* knockdown in mouse prostate, breast, and gastric cancer models (Chen et al., 2021; Liu et al., 2022; Wang et al., 2021).

The minichromosome maintenance protein complex (MCM) genes, such as *MCM2* and *MCM7*, have critical roles in growth and proliferation (Forsburg, 2004). *MCM2* knockdown diminished viability and increased G0/G1 arrest in ovarian cancer cells (Deng et al., 2019). *MCM7* inhibition led to a decrease in the esophagus (Qiu et al., 2017) and hepatocellular carcinomas (Qu et al., 2017) viability, colony-forming ability, and migration capacity due to reducing phosphorylation of AKT1 and mTOR proteins with decreasing *CDK1, CCNE1*, and *CCNE2* expression levels (Qiu et al., 2017). 14-3-3 proteins, also known as the YWHA family, consist of 7 isoforms that have isoform-specific functions (Eisa et al., 2019). The viability, clonogenicity, proliferation, and in vivo tumor enlargement of hepatocellular carcinoma cells were suppressed after *YWHAB* inhibition (Hu et al., 2020). Moreover, overexpression of the *YWHAB* increased viability and invasion while decreasing the apoptosis rate in cervical cancer cells (Zhang et al., 2021). Additionally, a recent report underlined YWHAH’s role in stimulating thyroid cancer cell proliferation and invasion (Zhou et al., 2020).

For HQ-treated SKOV-3 cells, RRM2, PSMD2, PSME2, COX2, COX4l1, and CDK6 proteins were the main low-abundant DA proteins enriching Reactome and KEGG proliferation-related pathways. RRM2 is essential for DNA replication and repair and is involved in cell cycle progress (Nordlund and Reichard, 2006). Migration and invasion of gastric (Zhong et al., 2016), and pancreas cancers (Duxbury and Whang, 2007) were enhanced through the overexpression of *RRM2* accompanied by raised MMP2 and MMP-9 levels, and activation of NF-KB and AKT signaling. This tumor-promoting effect of RRM2 was observed in multiple other studies. Decreased viability in neuroblastoma (Li et al., 2018, p. 2) and glioblastoma (Sun et al., 2019), and invasion capacity in breast carcinoma (Zhuang et al., 2020) cells by *RRM2* inhibition were defined previously.

In breast carcinoma cells, viability and clonogenicity were negatively affected upon *PSMD2* repression, cells were arrested in G0/G1 phase, and the level of the proteins involved in the progression of the cell cycle (CDK6 and CCND1) was significantly decreased (Li et al., 2018). Likewise, *PSMD2* knockdown reduced the proliferation rate, stimulated G0/G1 arrest, and increased apoptosis in HepG2 cells (Tan et al., 2019). There are contradictory data on the role of proteasome activator subunit 2 (PSME2) in cancer progression. In gastric cancer, knocked down *PSME2* enhanced viability, clonogenicity, and tumorigenicity (Huang et al., 2010; Zheng et al., 2012). On the other hand, in breast and lung cancer cells, *PSME2* downregulation reduced the viability, invasion, and migration (Li et al., 2019).

Cyclooxygenase family members (COX) are responsible for prostanoids’ production, inflammation, and pain metabolism (Rouzer and Marnett, 2009). Suppression of COX2 significantly reduced the viability and increased the apoptotic rate of breast carcinoma cells (McFadden et al., 2006). Furthermore, in COX2 inhibitor-treated murine model, decreased tumor development, incidence, and spontaneous metastasis were reported (Kundu et al., 2002). Overexpression of COX4l1 increased glioma cell viability, tumorigenicity, and neuronal stem cell markers level in vitro, and enhanced tumor growth and proliferating tumor cell number in vivo (Oliva et al., 2015). In lung adenocarcinoma cells downregulating *CDK6* inhibited the proliferation rate and stimulated G0/G1 arrest (Wu et al., 2010). Furthermore, inhibition of *CDK6* repressed tumor enlargement in the in vivo lung xenograft model (Zhu et al., 2013).

As discussed above, the roles of identified DA proteins were associated with cancer pathogenesis in previous studies. A significant decrease in the expression level of these genes in the HQ-treated breast or ovarian cancer cells supports the antineoplastic activity of HQ.

The construction of the PPI network was useful to explore the association of the identified proteins. Most SKOV-3 and MCF-7 DA proteins were connected with more than one protein in the query, which underlines the HQ impact on cooccurred and coexpressed proteins. Therefore, following the HQ treatment, a domino effect was probably observed for the MCF-7 and SKOV-3 proteome. DA protein PPI network for MCF-7 proteome unveiled RPS5 as one of the core proteins (Figure 6). A high-abundant protein in the HQ-treated MCF-7 cells, RPS5, acts as a tumor suppressor by regulating TP53 and c-myc, and its deficiency is associated with tumor progression and aggressiveness (Fancello et al., 2017). HSPA5, a master regulator of unfolded protein response, was high-abundant in HQ treated in SKOV-3 cells and was detected as one of the core proteins in the PPI network. An increased level of HSPA5 negatively regulates apoptosis (Cerezo and Rocchi, 2017) and ferroptosis (Chen et al., 2019), which may imply the high-stress conditions triggered by HQ.

Altogether, our data indicate the anticarcinogenic activity of HQ on breast and ovarian cancer cells. Although distinct sets of proteins were identified as differentially abundant in HQ-treated SKOV-3 or MCF-7 cells after proteomics analysis, assessment of deep bioinformatics results with cellular assays provided a causative link between altered biological processes and cell-level impact upon HQ treatment. For instance, the suppression of cell-cycle phase transition and cell-cycle-related pathways while the activation of apoptosis-related ones following the HQ treatment in SKOV-3 cells confirmed the observed cellular effects at the molecular level. On the other hand, changes in translation initiation and cytoplasmic translation pathways after the HQ treatment in MCF-7 cells, according to PPI network analysis, may explain the observed toxicity and decreased survival capacity. A relatively high average node degree detected in PPI analysis underlines the highly connected network of identified DA proteins, emphasizing the need to explore identified hub proteins’ roles in follow-up studies. Moreover, the different profiles of DA proteins in MCF-7 and SKOV-3 cells upon HQ administration might be due to the differential expression of potassium channels in breast and ovarian cancer cells. More research is required to dissect the mechanisms by which similar cellular effects were observed through diverse molecular routes in breast and ovarian cancers.

## 5. Conclusions

In summary, our experimentation findings revealed HQ’s anticancer activity on breast and ovarian cancer cells for the first time. HQ played growth-limiting and antimigrative roles by modulating the expression level of the genes required for cell division, DNA replication, DNA repair, matrix-remodeling, regulation of apoptosis, and stress response. Thus, this small molecule can be considered as a promising anticarcinogenic compound for further in vivo studies to evaluate its potential in preclinic trials. Future research on other cancer types is required to extend its potential usability in cancer treatment.

## Supplementary Information



















## Data Availability

The proteome data was deposited in MendeleyData. Supplementary data can be accessed at the following link: Demircan, Turan (2022), “Proteomics data of control and HQ-treated MCF7 and SKOV-3 cells”, Mendeley Data, V1, https://doi.org/10.17632/hd75n424fx.1
