# Supplementary material for: Hydroquinidine displays a significant anticarcinogenic activity in breast and ovarian cancer cells via inhibiting cell-cycle and stimulating apoptosis
Source: Turk J Biol. 2023 Jan 11;47(1):44–60. doi: 10.55730/1300-0152.2640 (PMC10388048; doi:10.55730/1300-0152.2640)
Supplement: Supplementary file 1 [file BIY-2209-4_1_Supplementary_File_S1.docx]

**Hydroquinidine Displays a Significant Anti-carcinogenic Activity in Breast and Ovarian Cancer Cells via Inhibiting Cell-cycle and Stimulating Apoptosis**

**Mervenur Yavuz, Betül Şahin, Ahmet Tarık Baykal, Turan Demircan**


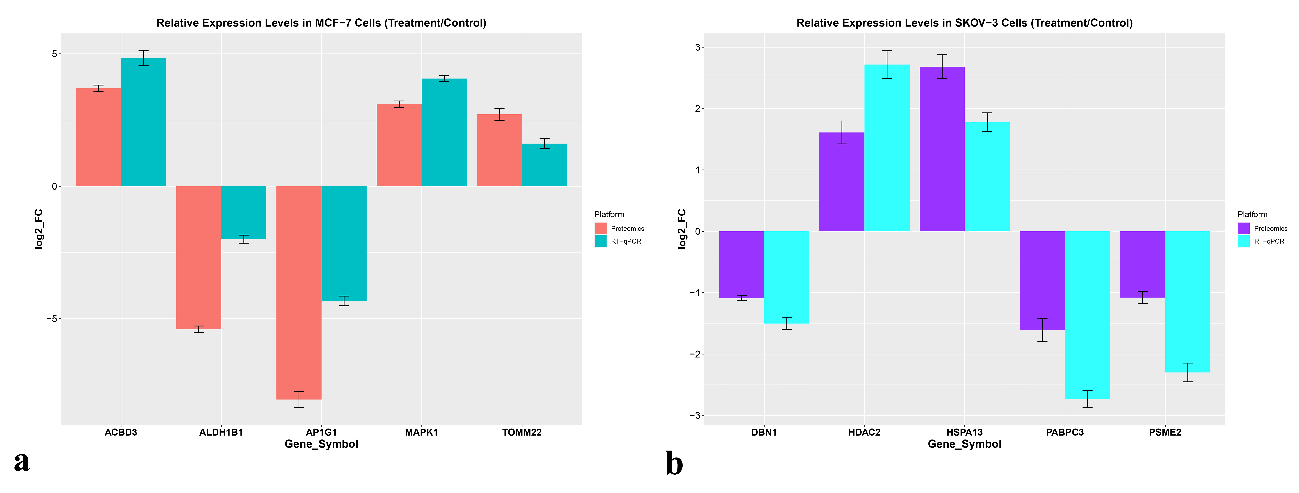


**Figure S1. Validation of proteomics data.** Proteomics data was validated by RT-qPCR. a) Relative expression levels of ACBD3, ALDH1B1, AP1G1, MAPK1, and TOMM22 genes and proteins in MCF-7 cells b) Relative DBN1, HDAC2, HSPA13, PABPC3, and PSME2 gene and protein expression levels of SKOV-3 cells.

**Table S9.** Primer list used in the RT-qPCR

| **Gene Symbol** | **Forward Primer** | **Reverse Primer** |
| --- | --- | --- |
| *ACBD3* | ctggaggagttgtacggcc | tgggcccataagaacttgct |
| *ALDH1B1* | gacatcccctacaaccagct | atcagcccggtcaccttc |
| *AP1G1* | gagctgatccggaccatcc | attttgccacattccgacatc |
| *DBN1* | tttccggccacttcgagaac | cagcttgggagtccttgacg |
| *GAPDH* | ggagcgagatccctccaaaat | ggctgttgtcatacttctcatgg |
| *HDAC1* | cgccctcacaaagccaatg | ctgcttgctgtactccgaca |
| *HSPA13* | tcggctgttttgactctcct | ccccaacagaacaataggtgg |
| *MAPK1* | tagcaagaacaacatccgagac | gctccaggcgacatattagg |
| *PABPC3* | tgactgaggcgatgctctac | ggtgatcaagtccctgcaga |
| *PSME2* | cttttccaggaggctgaggaat | agggaagtcaagtcagccac |
| *TOMM22* | ggacgacgatgaggagctag | tctgagccacaaagagggaa |
